# The Psychometric Properties of the Center for Epidemiologic Studies Depression Scale in Chinese Primary Care Patients: Factor Structure, Construct Validity, Reliability, Sensitivity and Responsiveness

**DOI:** 10.1371/journal.pone.0135131

**Published:** 2015-08-07

**Authors:** Weng Yee Chin, Edmond P. H. Choi, Kit T. Y. Chan, Carlos K. H. Wong

**Affiliations:** 1 Department of Family Medicine and Primary Care, The University of Hong Kong, 3/F, 161 Main Street, Ap Lei Chau Clinic, Ap Lei Chau, Hong Kong; 2 School of Nursing, The University of Hong Kong, 4/F, William M.W. Mong Block, 21 Sassoon Road, Pok Fu Lam, Hong Kong; Kings College, UNITED KINGDOM

## Abstract

**Background:**

The Center for Epidemiologic Studies Depression Scale (CES-D) is a commonly used instrument to measure depressive symptomatology. Despite this, the evidence for its psychometric properties remains poorly established in Chinese populations. The aim of this study was to validate the use of the CES-D in Chinese primary care patients by examining factor structure, construct validity, reliability, sensitivity and responsiveness.

**Methods and Results:**

The psychometric properties were assessed amongst a sample of 3686 Chinese adult primary care patients in Hong Kong. Three competing factor structure models were examined using confirmatory factor analysis. The original CES-D four-structure model had adequate fit, however the data was better fit into a bi-factor model. For the internal construct validity, corrected item-total correlations were 0.4 for most items. The convergent validity was assessed by examining the correlations between the CES-D, the Patient Health Questionnaire 9 (PHQ-9) and the Short Form-12 Health Survey (version 2) Mental Component Summary (SF-12 v2 MCS). The CES-D had a strong correlation with the PHQ-9 (coefficient: 0.78) and SF-12 v2 MCS (coefficient: -0.75). Internal consistency was assessed by McDonald’s omega hierarchical (ωH). The ωH value for the general depression factor was 0.855. The ωH values for “somatic”, “depressed affect”, “positive affect” and “interpersonal problems” were 0.434, 0.038, 0.738 and 0.730, respectively. For the two-week test-retest reliability, the intraclass correlation coefficient was 0.91. The CES-D was sensitive in detecting differences between known groups, with the AUC >0.7. Internal responsiveness of the CES-D to detect positive and negative changes was satisfactory (with p value <0.01 and all effect size statistics >0.2). The CES-D was externally responsive, with the AUC>0.7.

**Conclusions:**

The CES-D appears to be a valid, reliable, sensitive and responsive instrument for screening and monitoring depressive symptoms in adult Chinese primary care patients. In its original four-factor and bi-factor structure, the CES-D is supported for cross-cultural comparisons of depression in multi-center studies.

## Introduction

Depressive disorders are disabling impairing people’s functioning and health-related quality of life (HRQOL) [1]. At its worst, depressive symptoms can lead to suicide. Thus, the detection of depressive symptoms and provision of treatments are of paramount importance to diminish the negative impacts of depressive disorders on individuals and society as a whole.

The Center for Epidemiologic Studies Depression Scale (CES-D) is one of the more frequently used screening instruments for depressive symptoms. According to Shafer, the CES-D is a balanced and comprehensive instrument [2] and is the only instrument which assesses interpersonal aspects. The CES-D, which was developed by Radloff [3], has been widely used in different age groups including adolescents [4], adults [5], and the elderly [6]; and patient populations such as cancer patients [7] and patients with heart disease [8]. The CES-D has also been used in a variety of Chinese populations including Chinese in America [9], Chinese in Hong Kong [10], Chinese in Mainland China [11] and Chinese in Taiwan [12]. Despite its widespread use, the psychometric properties of the CES-D have only been tested in selective Chinese samples [13]. In the Hong Kong setting, previous studies examining the psychometric properties of the CES-D have used methods which limit its applicability and generalizability. One study incorporated a selected sample of married couples with sample size insufficient for the statistical methods applied [14]. A more recent study sampled school-aged Chinese adolescents [15] who may possess unique conceptualizations of depressive symptomatology due to the complexities of adolescence. In terms of translation, various locally developed versions of the CES-D exist, however those that have been published and used in adult samples have had weak conceptual equivalence to the original English version for modern Hong Kong Chinese [14, 16]. This has been further affected by the modification of response choices for the CES-D items when adapted for administration in Chinese. The original CES-D adopts a four-point scale, whilst many Chinese versions use a five-point scale and a different scoring rubric [14]. Discrepancies in translation and response option can threaten the validity and affect cross-cultural interpretability of findings [17, 18]. There is thus a need to validate a well-translated instrument, with good translational, conceptual and structural equivalence to the original CES-D in a wide sampling population.

The CES-D is widely used in longitudinal studies [19, 20]. Despite this, there is little published evidence for the instrument’s responsiveness (ability to detect change over time). An instrument that is not responsive can lead to false negative results [21, 22]. Establishing the responsiveness of the CES-D can strengthen the rationale for using it in longitudinal studies.

### Aim and objectives

The aim of this study was to validate the CES-D for use in Chinese primary care patients in Hong Kong by examining the factor structure, construct validity, reliability, sensitivity and responsiveness.

## Methods

This study was conducted as part of an epidemiological study to examine the natural history of depressive disorders in Hong Kong's primary care. The study protocol is published [23].

### Design

A 12-month longitudinal observational study was conducted on patients recruited through a primary care practice-based research network.

### Sampling and participant

Fifty nine primary care doctors working in public and private sector clinics territory wide across Hong Kong were recruited using the mailing list of the Hong Kong College of Family Physicians. All eligible patients presenting to the study doctor on one randomly selected day each month between were invited to join the study. All patients consulting the study doctor (for any reason) were consecutively approached by field workers in the waiting room to join the study. Exclusion criteria were (1) aged < 18 years, (2) had cognitive or communication difficulties (3) had already been recruited to the study and (4) not having a face-to-face consultation with the doctor. Subjects were asked to self-complete a baseline questionnaire containing items on socio-demography, the PHQ-9, the CES-D and the Short Form-12 Health Survey version 2 (SF-12 v2). If subjects had difficulty completing the questionnaire due to visual impairment or poor literacy, the field worker helped to administer the questionnaire. All subjects completing the baseline survey were invited to participate in the longitudinal study. Those who consented by providing their name and contact number were followed by telephone interview at 2 weeks (for evaluating test-retest reliability, only administered to those who screened PHQ-9 positive) and 12 weeks (for evaluating responsiveness). Follow-up questionnaires contained of the CES-D, the PHQ-9 and the SF-12 v2. Data was collected between November 2012 and January 2014.

### Ethics approval

This study was approved by the Institutional Review Board of the University of Hong Kong/Hospital Authority Hong Kong West Cluster, the Research Committee of Hong Kong Sanatorium and Hospital, the Research Ethics Committee for Hong Kong Hospital Authority Kowloon East and Kowloon Central Clusters, the Joint Chinese University of Hong Kong—New Territories East Cluster Clinical Research Ethics Committee, the Ethics Committee of the Matilda International Hospital, and the Research Committee of the Evangel Hospital.

### Study instruments

#### The Centre for Epidemiologic Studies Depression Scale (CES-D)

The CES-D consists of twenty questions which measures depressive symptomatology during the past week. Respondents rate the frequency of occurrence of each symptom on a 4-point Likert scale (0: less than 1 day; 1: last for 1–2 days; 2: last for 3–4 days; and 3: last for 5–7 days). The scores for each item can be summed to give a total score ranging from 0 to 60 with higher scores indicating more severe depression. Based on the total score, patients can be categorized as having mild depression (score 16 to 26) or major depression (score 27 to 60). The Chinese version of the CES-D used in this study was adopted from the translation used in the Central and Western District Adolescent Health Survey in Hong Kong [15, 24]. In the earlier study the authors used 5-point response scale, which differed from Radloff’s original questionnaire [3]. For this current study, a 4-point response option was used in line with original CES-D. The final Chinese CES-D used for this current study had the translational and conceptual equivalence confirmed by a bilingual family medicine specialist and a bilingual registered nurse. The instrument version used is available in **S1 Instrument**.

#### The Patient Health Questionnaire 9 (PHQ-9)

The PHQ-9 consists of nine questions, based on the criteria for the diagnosis of major depressive disorder in the Diagnostic and Statistical Manual of Mental Disorders, Fourth Edition (DSM-IV) [25]. Subjects were asked to indicate the frequency of occurrence for each symptom over the past two weeks on a 4-point Likert scale (0: not at all; 1: several days; 2: more than half the days; and 3: nearly every day) [25]. The scores of the nine questions are summed to give a total score ranging from 0 to 27, with higher scores indicating more severe depressive symptoms. Based on the total score, patients can be categorized as having minimal depression (score 1–4), mild depression (score 5–9), moderate depression (score 10–14), moderately severe depression (score 15–19) or severe depression (score 20–27). The PHQ-9 is responsive [26] and has been translated and validated in Hong Kong primary care patients [27] and in the Hong Kong general population [28]. In this study, the PHQ-9 was used to assess the convergent validity of the CES-D as they are both depression instruments, measuring a similar construct; and to capture the change in depression severity at the 2-week and 12-week follow-up interviews.

#### The SF-12 Health Survey Version 2.0 (SF-12 v2)

The SF-12 v2 is a generic HRQOL measure, which generates two summary scores, namely physical and mental component summary scores (PCS and MCS) with higher scores indicating better HRQOL [29]. The SF-12 v2 has been translated and validated for use in the Hong Kong’s primary care setting [30]. It has been proposed that the SF-12 v2 MCS can be used as a depression screening tool in the general population [31]. Therefore, in this study, the SF-12 v2 MCS was also used to assess the convergent validity of the CES-D.

### Statistical analysis

#### Floor and ceiling effect

Descriptive statistics (mean and standard deviation) and the percentages of floor and ceiling of the CES-D, the PHQ-9 and the SF-12 v2 MCS scores were calculated. 15% was used as the threshold for a significant floor or ceiling effect [32].

#### Factor structure

A comparison of three different CES-D factor structure models was conducted: a four-factor model (as proposed by Radloff [3]), a second-order factor model [33], and a bi-factor model [33]. For a four-factor model, it is proposed that the CES-D has four factors, namely depressed affect, positive affect, somatic and retarded activity and interpersonal problems. For a second-order factor model, there is a single second-order general depression factor to explain the covariance among the four first-order factors. In a bi-factor model, the general depression factor has no correlation with the four specific factors. In other words, the general depression factor explains the covariance among all scale items of the CES-D, while the specific factors explains the variance of the items within the specific factors [33].

Confirmatory factor analysis (CFA) models for ordinal data were performed using a polychoric correlation matrix to confirm the proposed models and to compare the goodness of fit between different models. Standard maximum likelihood extraction on polychoric correlation matrix was used. The goodness-of-fit statistics of the model were assessed using standardized root mean square residual (SRMR), root mean square error of approximation (RMSEA), comparative fit index (CFI) and Tucker-Lewis index (TLI) as recommended by Hu and Bentler [34]. Model fit was considered as good if the value of the SRMR was close to or below 0.08 [34], the value of the RMSEA was close to or below 0.06 [34, 35], and the values of the CFI and the TLI were greater than 0.9 (>0.90 acceptable, >0.95 excellent) [34, 36]. For model comparison, a significant chi-square difference (∆χ2) and the change in CFI (ΔCFI) >0.01 indicated that two models were significantly different.

#### Construct validity

Internal construct validity was assessed by examining the item-total correlation corrected for overlap using a correlation coefficient ≥0.4 as the cut-off for adequate correlation [37]. Convergent validity was assessed by computing Person’s correlations between the CES-D, the PHQ-9 and the SF-12 v2 MCS. It was hypothesized that the CES-D score would have a stronger correlation with the PHQ-9 score than with the SF-12 MCS score because both CES-D and PHQ-9 specifically measure depressive symptoms whilst the SF-12 MCS was designed to measure mental health-related quality of life.

#### Reliability

The internal consistency of the CES-D was assessed by McDonald’s omega hierarchical (ωH). This method is recommended for a scale that has a hierarchical factor structure. Test-retest reliability was assessed by examining the intra-class correlation coefficient (ICC) in subjects who had no change in PHQ-9 score between the baseline and 2-week testing. An ICC ≥ 0.7 was used to indicate good test-retest reliability [32].

#### Sensitivity

The sensitivity of the CES-D to discriminate between subjects with doctor-diagnosed depression and subjects without doctor-diagnosed depression was assessed by known-group comparison and by calculating the area under a receiver operating characteristic (ROC) curve [38]. Study doctors who were blinded to the PHQ-9 and CES-D screening scores were asked to document on a case record form whether they felt the patient had a clinically significant depressive symptoms based on their clinical judgment, without using any depression screening tools. Independent t-test was used to compare the mean CES-D scores between groups. Cohen’s d effect size was also calculated. It was hypothesized that subjects with doctor-detected depression would have a higher CES-D score than those without. The area under a ROC curve (AUC) can show the probability that an instrument correctly classifies patients according to an external criterion. For this study, the external criterion for assessing sensitivity was based on the doctor’s clinical judgment on whether the subject had clinically significant depressive symptoms or not. The value of AUC is typically between 0.5 and 1.0, with 1.0 representing perfect discriminatory power whilst 0.5 representing no discriminatory power. A sensitive instrument should have AUC value ≥ 0.7 [32]. The AUC of the CES-D and the PHQ-9 and their 95% confidence intervals were calculated. It was hypothesized both CES-D and PHQ-9 would be able to discriminate between patients with doctor-diagnosed depression and those without, with an AUC >0.7.

#### Responsiveness

Two different approaches can be used to evaluate the responsiveness of an instrument. Internal responsiveness is the ability of an instrument to detect change over a pre-specified time frame. External responsiveness is the ability of an instrument to detect a clinically important change relating to the corresponding change in a reference measure of health status [21, 22, 39, 40].

To assess the internal responsiveness of the CES-D, subjects were divided into three groups according to their change in PHQ-9 scores between baseline and 12-weeks, namely (1) improved depressive symptoms (i.e. reduced PHQ-9 score), (2) stable depressive symptoms (i.e. same PHQ-9 score) or (3) worsened depressive symptoms (i.e. increased PHQ-9 score). For each group, changes in the mean scores of both the CES-D and the SF-12 MCS between baseline and 12-week interviews were examined by paired t-test. The differences in CES-D scores between baseline and 12-weeks were evaluated by the standardized effect size (SES) [41], the Cohen’s d effect size (ES) [42] and the standardized response mean (SRM) [43]. Since the most appropriate effect size for calculating responsiveness statistics remains controversial, three effect sizes were used [44]. The effect size statistics can provide a clear interpretation of the magnitude of the change of the PHQ-9 score in each group. The values of SES, ES and SRM were interpreted as trivial (<0.2), small (≥0.2 and <0.5), moderate (≥0.5 and <0.8) and large (≥0.8), according to Cohen [42] and Liang [43]. Internal responsiveness was supported if the difference was ≥0.2. It was hypothesized that 1) the CES-D score would be decreased with effect size ≥0.2 in the improved group; 2) there would be no statistically significant changes in the CES-D scores in the stable group; and 3) the CES-D score would be increased in the worsened group with effect size ≥0.2. It was also hypothesized that the CES-D would be more responsive than the SF-12 v2 MCS.

For assessing external responsiveness, subjects were divided into two groups according to the change of the PHQ-9 score between baseline and 12-weeks, namely improved depressive symptoms (i.e. decreased PHQ-9 score) and stable/worsened depressive symptoms (i.e. same/increased PHQ-9 score). External responsiveness was determined by comparing the change in CES-D mean scores between groups by independent t-test and by the ROC curve analysis [44]. The AUC of the CES-D and SF-12 MCS and the 95% confidence intervals were calculated. The ROC curve provides an overview of the relationship between a measure and an external criterion of change. Conceptually, AUC represents the probability of a random patient with improved depressive symptoms to have a larger improvement in score than a random patient with stable/worsened depressive symptom, with a value = 0.5 representing no discriminatory power, and a value = 1 representing perfect discriminatory power. A value ≥0.7 was used as the threshold of good discriminatory power [45]. It was hypothesized that the AUC of the CES-D would be >0.7; and the CES-D would be more externally responsive than the SF-12 v2 MCS.

Data analyses were conducted using LISREL (version 8.80 for Windows) for factor analysis and SPSS (version 20.0 for Windows) for other statistical tests.

## Results

Baseline characteristics of the subjects are shown in **Table 1.** After excluding subjects with missing values in the PHQ-9, CES-D or the SF-12, a total of 3686 subjects were included for the evaluation of the psychometric properties of the CES-D. Subjects mean age was 49.4 years and 58.1% were female. All respondents were of Chinese ethnicity. The subject recruitment flow chart is shown in **S1 Fig**.

**Table 1:** Descriptive statistics of the CES-D, PHQ-9 and the SF-12 v2 MCS and Socio-demographic characteristics of study subjects (n = 3686).

**Scale score**			
		Floor	Ceiling
Mean CES-D (SD)	9.5 (9.3)	12.9%	0.0%
Mean PHQ-9 (SD)	4.4 (4.4)	18.8%	0.0%
Mean SF-12 v2 MCS (SD)	53.0 (11.2)	0.0%	0.0%
**Socio-demographics**			
Gender (n, %)			
Male	1,531 (41.5%)		
Female	2,140 (58.1%)		
Missing	15 (0.4%)		
Mean age (SD)	49.4 (17.3)		
Age group (n, %)			
18–24 years	247 (6.7%)		
25–34 years	647 (17.6%)		
35–44 years	619 (16.8%)		
45–54 years	675 (18.3%)		
55–64 years	696 (18.9%)		
≥ 65 years	757 (20.5%)		
Missing	45 (1.2%)		
Education level (n, %)			
No formal school	233 (6.3%)		
Primary	675 (18.3%)		
Secondary	1,585 (43.0%)		
Tertiary	1,185 (32.1%)		
Missing	8 (0.2%)		
Marital status (n, %)			
Single	934 (25.3%)		
Married	2,322 (63.0%)		
Widow(er)	283 (7.7%)		
Separated or divorced	137 (3.7%)		
Missing	10 (0.3%)		
Employment status (n, %)			
Working	2,260 (61.3%)		
Not Working	1,417 (38.4%)		
Missing	9 (0.2%)		
Monthly household income (n, %)			
≤ $5,000	473 (12.8%)		
$5,001–10,000	297 (8.1%)		
$10,001–20,000	696 (18.9%)		
$20,001–30,000	623 (16.9%)		
$30,001–40,000	442 (12.0%)		
> $40,000	839 (22.8%)		
Missing	316 (8.6%)		

CES-D: the Center for Epidemiologic Studies Depression Scale

PHQ-9: the Patient Health Questionnaire-9

SD: standard deviation

SF-12 v2 MCS: the Short Form-12 Health Survey version 2 Mental Component Summary

### Floor and ceiling effect

The descriptive statistics of the CES-D, PHQ-9 and SF-12 v2 MCS scores at baseline interview are shown in **Table 1.** 12.9% and 18.8% of subjects achieved minimum CES-D and PHQ-9 scores, respectively whilst no subject achieved the maximum CES-D or PHQ-9 score.

### Factor structure

Results of the CFA are shown in **Table 2**, **Table 3**and **S2 Fig**. For all three models, the values of the SRMR were well below 0.08 whilst the values of the RMSEA were below 0.06. The values of CFI and TLI were greater than 0.90. Among the three models tested, although Radloff’s original proposed four-factor structure was acceptable, the bi-factor model had a better fit, with a smaller value of SRMR and RMSEA, and a larger value of CFI and TLI. In the bi-factor model, with the exception of the four “positive affect” items and two “interpersonal problem” items, all other items had a higher factor loading on “general factors” than on the corresponding specific factors.

**Table 2:** Factor structure and internal construct validity of the CES-D.

		Mean (SD) (n = 3686)	Corrected item-total score correlation^	Factor loading	Factor loading
Somatic				Somatic	General
1	I was bothered by things that usually don’t bother me	0.39 (0.74)	0.54	0.073	0.588
2	I did not feel like eating; my appetite was poor	0.28 (0.66)	0.42	0.243	0.432
5	I had trouble keeping my mind on what I was doing	0.41 (0.77)	0.57	0.450	0.559
7	I felt that everything I did was an effort	0.37 (0.75)	0.56	0.389	0.559
11	My sleep was restless	0.90 (1.12)	0.33	0.166	0.380
13	I talked less than usual	0.39 (0.73)	0.52	0.149	0.529
20	I could not get "going"	0.51 (0.83)	0.66	0.361	0.672
Depressed affect				Depressed affect	
3	I felt that I could not shake off the blues even with help from my family	0.27 (0.65)	0.61	0.097	0.672
6	I felt depressed	0.45 (0.77)	0.72	-0.240	0.881
9	I thought my life had been a failure	0.30 (0.69)	0.52	0.215	0.563
10	I felt fearful	0.29 (0.65)	0.55	0.235	0.595
14	I felt lonely	0.34 (0.73)	0.60	0.209	0.658
17	I had crying spells	0.14 (0.47)	0.45	0.144	0.500
18	I felt sad	0.53 (0.82)	0.69	-0.050	0.802
Positive affect				Positive affect	
4	I felt that I was just as good as other people	0.98 (1.24)	0.25	0.559	-0.124
8	I felt hopeful about the future	0.79 (1.11)	0.43	0.637	-0.300
12	I was happy	0.88 (1.03)	0.60	0.557	-0.520
16	I enjoyed life	0.77 (1.04)	0.57	0.617	-0.466
Interpersonal problems				Interpersonal problems	
15	People were unfriendly	0.27 (0.61)	0.45	0.629	0.441
19	I felt that people disliked me	0.22 (0.54)	0.50	0.715	0.480

CES-D: the Center for Epidemiologic Studies Depression Scale. SD: Standard deviation.

^ The correlation between each item and the total CES-D score that excluded that item

**Table 3:** Goodness-of-fit statistics of each model and model comparison.

**Goodness-of-fit**							
Model	df	χ2	Relative χ2	SRMR	RMSEA	CFI	TLI
1. Four factor	164	1964.588	11.98	0.0411	0.0546	0.980	0.976
2. Second-order factor	166	1968.140	11.86	0.0412	0.0543	0.980	0.977
3. Bi-factor	144	981.654	6.84	0.0242	0.0397	0.990	0.987
**Model comparison**							
Model	Δdf	Δχ2	P-value	ΔCFI			
1–2	2	3.552	0.169	0.000			
2–3	22	986.486	<0.001	-0.010			
1–3	20	982.934	<0.001	-0.010			

df = degree of freedom; SRMR = standardized root mean square residual; RMSEA = root mean square error of approximation; CFI = comparative fit index; TLI = Tucker–Lewis index

### Construct validity

The results of the analyses to evaluate internal construct validity are shown in **Table 2**. The item-total correlations corrected for overlap were >0.4 for all items, except for item 4 (0.25) and item 11 (0.33). The Pearson’s correlation coefficients are shown in **Table 4**. The CES-D total score had a strong correlation with the PHQ-9 total score (r = 0.78) and the SF-12 v2 MCS score (r = -0.75). The construct validity of the CES-D was supported.

**Table 4:** Convergent validity, reliability and sensitivity of the CES-D.

**Convergent validity**									
**Pearson's Correlation (n = 3686)**									
			PHQ-9			SF-12 v2 MCS			
CES-D			0.78 ^^			-0.75 ^^			
PHQ-9						-0.65 ^^			
**Reliability**									
**Internal consistency (n = 3686)**									
**McDonald’s omega hierarchical (ωH)**									
General depression			0.855						
Somatic			0.434						
Depressed affect			0.038						
Positive affect			0.738						
Interpersonal problems			0.730						
**Distribution of change of mental health at 2-week interview (n = 383)**									
Worsened mental health (n, %)			80 (20.9%)						
Stable mental health (n, %)			58 (15.1%)						
Improved mental health (n, %)			245 (64.0%)						
**2-week test-retest reliability** ^#^									
Intraclass correlation coefficient *(n = 58)			0.91						
**Sensitivity (n = 3521)** ‡									
	No depression	Depression							
	n = 3257	n = 264			Cohen’s d				
	Mean (SD)	Mean (SD)		P-value ^+^	Effect Size		AUC		(95% CI)
CES-D	8.80 (8.51)	19.53 (13.14)		<0.01	0.97		0.750		(0.72, 0.78)
PHQ-9	4.05 (4.03)	8.98 (6.20)		<0.01	0.94		0.747		(0.71, 0.78)
SF-12 v2 MCS	53.70 (10.42)	42.31 (15.17)		<0.01	0.88		0.724		(0.69, 0.76)

AUC: the area under a receiver operating characteristic curve. CES-D: the Center for Epidemiologic Studies Depression Scale. CI: confidence interval. CES-D: the Center for Epidemiologic Studies Depression Scale. Cohen’s d effect size = (μ_Followup_- μ_Baseline_)/σ_pooled_. PHQ-9: the Patient Health Questionnaire-9. SD: standard deviation. SF-12 v2 MCS: the Short Form-12 Health Survey version 2 Mental Component Summary.

‡ 165 subjects had missing data.

^^ Correlation is significant at the 0.01 level (2-tailed).

^#^Only subjects with stable mental health (n = 58) were included in the assessment of test-retest reliability.

*Two-way random model

+ Independent t-test was used.

### Reliability

The results of the analyses to evaluate internal consistency and test-retest reliability are shown in **Table 4**. The ωH value for the general depression factor was 0.855. The ωH valus for “somatic”, “depressed affect”, “positive affect” and “interpersonal problems” were 0.434, 0.038, 0.738 and 0.730, respectively.

383 subjects were successfully contacted 2-weeks after the baseline interview. Test-retest reliability was assessed in 58 subjects (15.1%) who had no change in their PHQ-9 score over the 2-week period. The ICC of the CES-D was 0.91. The reliability of the CES-D was supported.

### Sensitivity

The results of the analyses to examine sensitivity to differentiate between subjects with depression and those without depression are shown in **Table 4**. The prevalence of doctor diagnosed depression was 7.50%. Statistically significant differences were detected between the two groups by the CES-D (effect size 0.97), the PHQ-9 (effect size 0.94) and the SF-12 v2 MCS (effect size 0.88). Furthermore, the CES-D, PHQ-9 and SF-12 v2 MCS were sensitive enough to detect differences between subjects, with an AUC >0.7 for all instruments. Among these three instruments, the CES-D had the largest AUC (0.75) confirming the sensitivity of the CES-D. The ROC curve for the sensitivity analysis shows in **S2 Fig**.

### Responsiveness

The results of the analyses to evaluate internal responsiveness are shown in **Table 5**. The groupings were based on the PHQ-9 scores. The CES-D total score reduced significantly (i.e. symptom improvement) in subjects with reduced depressive symptoms, with Cohen’s d effect size and SRM >0.8. The SF-12 v2 MCS also detected a statistically significant improvement in those subjects but the effect size statistics of the SF-12 v2 MCS were smaller than those of the CES-D. Moreover, both CES-D and SF-12 v2 MCS had statistically significant improvements in subjects whose PHQ-9 score had no change. Compared with patients with improved depressive symptoms, the effect size statistics of the CES-D and SF-12 v2 MCS were smaller in patients with stable depressive symptoms. The CES-D detected a statistically significant deterioration in subjects with worsened PHQ-9 score with all effect size statistics >0.2. On the contrary, the SF-12 v2 MCS could not detect any statistically significant differences in patients with worsened PHQ-9 scores.

**Table 5:** The responsiveness of the CES-D and the SF-12 v2 MCS.

**Internal responsiveness**							
	Mean (SD) at baseline	Mean (SD) at discharge	P-value^#^	Mean Change (SD)	Standardized effect size	Cohen's d effect size	Standardized response mean
Improved depressive symptoms, n = 1,420 (57.63%)							
CES-D	10.65 (8.94)	4.47 (6.15)	<0.01	6.18 (7.18)	0.69	0.81	0.86
SF-12 v2 MCS	51.60 (11.11)	56.87 (8.04)	<0.01	5.27 (9.70)	0.47	0.54	0.54
Stable depressive symptoms, n = 563 (22.85%)							
CES-D	3.87 (4.89)	2.46 (4.10)	<0.01	1.41 (4.53)	0.29	0.31	0.31
SF-12 v2 MCS	59.00 (7.06)	60.16 (6.11)	<0.01	1.16 (6.75)	0.16	0.18	0.17
Worsened depressive symptoms, n = 481 (19.52%)							
CES-D	7.38 (8.29)	9.22 (9.81)	<0.01	1.84 (7.69)	0.22	0.20	0.24
SF-12 v2 MCS	54.66 (10.63)	54.93 (10.72)	0.55	0.27 (9.90)	0.03	0.03	0.03
**External responsiveness**							
	Mean difference		(95% CI)		AUC	(95% CI)	
CES-D	6.27		(5.72, 6.82)		0.75	(0.73, 0.77)	
SF-12 v2 MCS	4.52		(3.78, 5.25)		0.64	(0.61, 0.66)	

AUC: the area under a receiver operating characteristic curve. CES-D: the Center for Epidemiologic Studies Depression Scale. Cohen’s d effect size = (μ_Followup_- μ_Baseline_)/σ_pooled._ CI: confidence interval. PHQ-9: the Patient Health Questionnaire-9. SD: standard deviation. SF-12 v2 MCS: the Short Form-12 Health Survey version 2 Mental Component Summary. Standardized effect size = (μ_Followup_- μ_Baseline_)/σ_Baseline._Standardized response mean = (μ_Followup_- μ_Baseline_)/σ_Followup-Baseline._ Stable depressive symptoms (same PHQ-9 score). Improved depressive symptoms (reduced PHQ-9 score). Mean difference: the difference in mean change between two groups (improved depressive symptoms vs. stable/worsened depressive symptoms). Worsened depressive symptoms (increased PHQ-9 score).

^#^ Paired t-test was used.

The results of the analyses assessing the external responsiveness are shown in **Table 5**. The differences in the mean change between the improved and stable/ worsened groups were statistically significant for the CES-D and the SF-12 v2 MCS. With a cut-off AUC>0.7, the CES-D (AUC = 0.75) but not the PHQ-9 (AUC = 0.64) was adequate to differentiate subjects who improved and those with stable or worsened depressive symptoms. The ROC curve for external responsiveness is shown in **S2 Fig**.

## Discussion

Our analyses confirmed that the CES-D is valid for use amongst Chinese adult primary care patients in Hong Kong. Although the best fitting factor model was the bi-factor model, Radolff’s four-factor model was also satisfactory. Our findings help to strengthen the rationale for using the CES-D to screen for depressive symptoms, to monitor disease progression, and that the instrument is valid for use in cross-cultural comparative studies.

### Factor structure

Our comparison of three competing factor structure models found that although the original four factor model was adequate, the data set fit better into a bi-factor model. The general depression factor was more dominant than other specific factors, particularly for “somatic complaints” and “depressed affects”. It has been suggested that the “positive affect” items are not part of the general depression factor and that a total CES-D score should be summed without the positive affect items. The positive affect items should instead be added together to generate a subscale score [14]. Despite a satisfactory model fit, both the “positive affect” and “interpersonal problems” items may not be part of the “general depression” factor as the items of these two domains had higher factor loading on the corresponding factors. Based on this, we suggest that if a bi-factor model is to be used, that the item scores for “somatic complaints” and “depressed affect” can be added together to generate a summary score, whilst two individual summary scores for “positive affect” and “interpersonal problems” can be generated respectively

### Construct validity

In the analysis of the item-total correlation, two question items (item 4: ‘Feeling as good as others’ and item 11: ‘Restless sleep’) did not reach the recommended cut-off point of 0.4, suggesting that the responses to these items may be less related to the other indicators of depressive symptoms. Furthermore, the mean scores of these items were much higher than the mean scores of most other CES-D items, which might lead to poorer correlations. Other studies have also reported low item-total correlations for these two items [46, 47]. In the Hong Kong context, item 4 could easily be interpreted as a comparison of general living standards, while item 11 could potentially be misinterpreted as sleep deprivation due to the engagement of bed-time social activities, work-related stress, ageing, etc.

The hypothesized correlations between the CES-D and other depression instruments were generally observed confirming its convergent validity. The CES-D total score correlated strongly with both the PHQ-9 total score and the SF-12 v2 MCS score, however it appears that the SF-12 v2 MCS had a stronger correlation than the PHQ-9. Our findings were similar to the results of a previous study which found that both CES-D (r = -0.76) and PHQ-9 (r = -0.68) had a strong correction with the SF-36 MCS, and when compared with the PHQ-9, the CES-D had a stronger correlation with the SF-36 MCS [48]. It is possible that the CES-D contains more items, which might lead to a higher correlation with the SF-12 v2 MCS.

### Reliability

The internal consistency for “general depression”, “positive affect” and “interpersonal problems” were supported, suggesting the use of subscale scores for these domains may be possible. However, the values for “somatic” and “depressed affect” were relatively low. Our findings were similar to those found by Gomez and McLaren, which found the acceptable internal consistency of the general factor and the “positive affect” domain [33]. The test-retest reliability of the CES-D in our population was reassuring and performed better than in other populations [49, 50].

### Sensitivity

The CES-D was sufficiently sensitive to differentiate patients with depressive symptoms from those without, and comparable to that of the PHQ-9 and the SF-12 v2 MCS.

### Responsiveness

The CES-D was responsive to both positive changes and negative changes in depressive symptoms as measured by the PHQ-9. However, it should be interpreted with caution because a positive change (improvement) was also detected within the stable group. The CES-D might be too responsive picking up “noises” [51, 52] which may not be clinically meaningful. Our findings suggest that the CES-D is a better instrument for longitudinal monitoring of depressive symptoms than the SF-12 v2 MCS.

### Clinical and research implications

Clinicians in primary care such as family doctors and nurse practitioners might not have specialized knowledge in diagnosing depression. Using the CES-D can help them to identify patients with depression in order to provide interventions or a prompt referral. Furthermore, the CES-D can be used for longitudinal monitoring and to evaluate the impact of treatment. In research, the CES-D can be used to estimate the prevalence, remission and relapse, to measure the severity of depressive symptoms, to screen for eligible patients for subject recruitment, and to evaluate effectiveness in intervention studies. Knowledge of the psychometric properties and evidence for the validity of the instrument in this setting assists in data interpretation and strengthens the rationale for its use in cross-cultural comparative studies.

### Limitations

As in other practice-based research studies, limitations existed for practical reasons. The baseline data was collected either through self-completion or face-to-face interview. In the case of the latter, items were not necessarily administered verbatim in all subjects, and the pre-set order was not always strictly followed. Such adjustments albeit deviated from the instruction of the original questionnaire were deemed essential during data collection as most of the study practices had fairly high caseload (20–40 patients per half-day session) and hence a challenge to administer 20 items in a short period of time. Also many patients were elderly and of relatively low educational status and hence the questionnaire was on occasion administered in a less structured manner, to allow better comprehension and completion of the survey. This lack of standardized instrument administration can potentially result in variations of item scores, and affect the reliability results and the factor structure obtained.

In this study, depression identification was not based on a structured clinical interview or made by psychiatrists, but by our study doctors in the setting of a general medical primary care consultation. Most of the study doctors were trained Family Medicine physicians, and all were familiar with the diagnostic criteria for depression, however, variations in the identification rate for depression by doctors can potentially affect the sensitivity analysis.

As we only included local primary care patients as our study subjects, this may preclude the generalizability of the validation results to secondary care patients who may have a more severe spectrum of depressive symptoms.

## Conclusions

This study found that the CES-D is a valid and reliable instrument to assess and monitor depressive symptoms in adult Chinese primary care patients. The original four-factor structure of the CED-S was applicable in our study population; however a bi-factor model appears to have a better fit. The CES-D was sensitive enough to screen for depression and was internally and externally responsive. It outperformed the SF-12 v2 MCS in capturing change overtime. We hope the instrument can be applied for Chinese in the worldwide diaspora.

## Supporting Information

S1 Appendix The bi-factor structure of the CES-D by confirmatory factor analysis. (PDF)

S1 Fig Subject recruitment flowchart. (PDF)

S2 Fig The sensitivity of the CES-D and the PHQ-9 to differentiate subjects with depression and those without depression. The CES-D and PHQ-9 were sensitive enough to detect difference between the subject, with an AUC >0.7 for all instruments.
(PDF)

S3 Fig The external responsiveness of the CES-D and the SF-12 v2 MCS. With the standard of the AUC>0.7, the CES-D (AUC = 0.75) but not the PHQ-9 (AUC = 0.64) was adequate to differentiate subjects who improved and those with stable or worsened depressive symptoms.
(PDF)

S1 Instrument The Center for Epidemiologic Studies Depression Scale (CES-D)-Chinese Version with English Translation. (PDF)
