# Supplementary material for: The Psychometric Properties of the Center for Epidemiologic Studies Depression Scale in Chinese Primary Care Patients: Factor Structure, Construct Validity, Reliability, Sensitivity and Responsiveness
Source: PLoS One. 2015 Aug 7;10(8):e0135131. doi: 10.1371/journal.pone.0135131 (PMC4529142; doi:10.1371/journal.pone.0135131)
Supplement: S1 Appendix — (PDF) [file pone.0135131.s001.pdf]

S1 Appendix

The bi-factor structure of the CES-D by confirmatory factor analysis

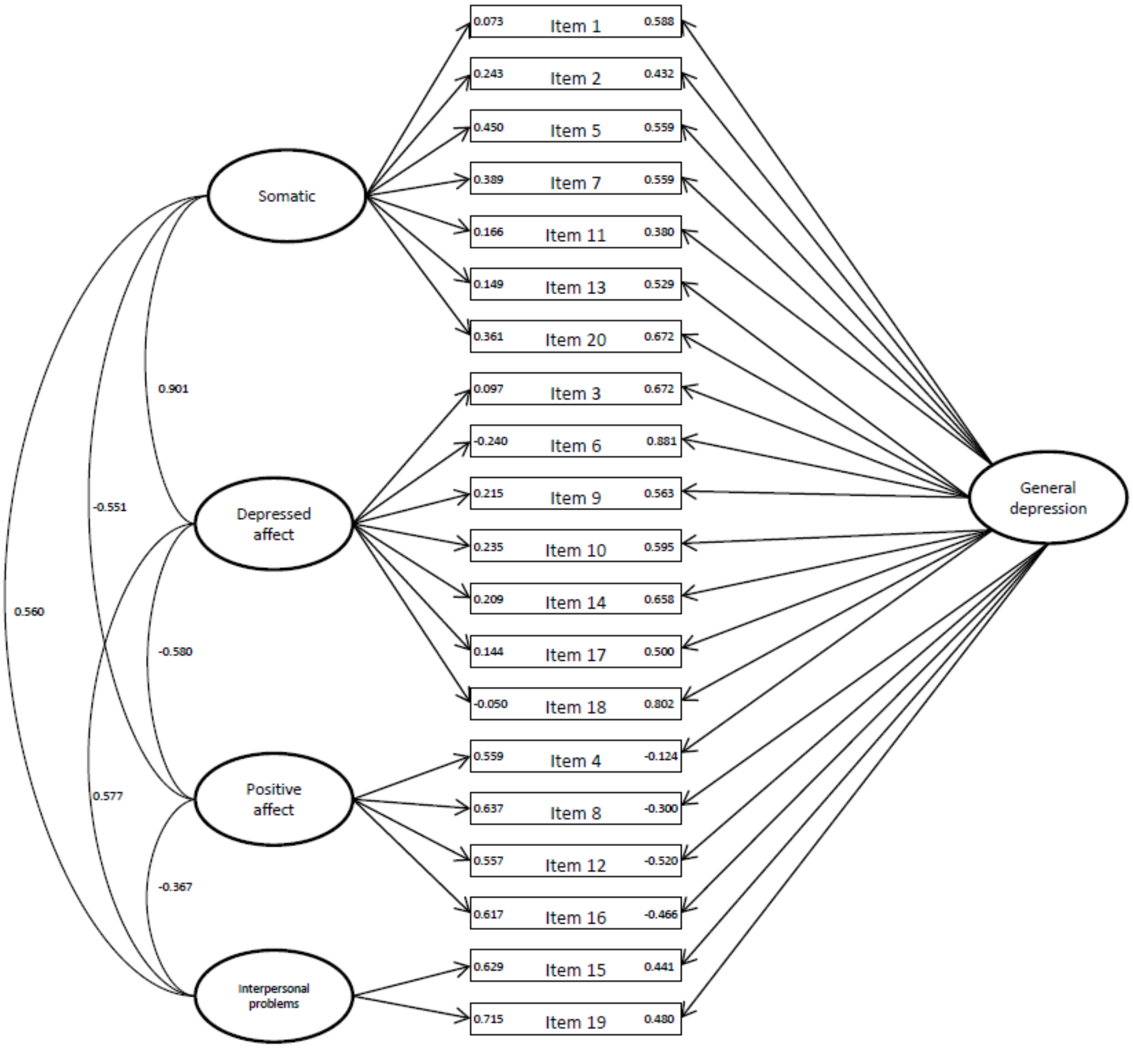

The figure shows the results of the confirmatory factor analysis by LISREL (version 8.80). In the bi-factor model, with the exception of the four "positive affect" items and two "interpersonal problem" items, all other items had a higher factor loading on the "general factors" than on the corresponding specific factors.

Correlation coefficient matrix of the 20 items of the CES-

	item 1	item 2	item 3	item 4	item 5	item 6	item 7	item 8	item 9	item 10	item 11	item 12	item 13	item 14	item 15	item 16	item 17	item 18	item 19	item 20
**item 1**	1																			
**item 2**	0.296	1																		
**item 3**	0.435	0.307	1																	
**item 4**	-0.081	-0.048	-0.097	1																
**item 5**	0.373	0.364	0.374	-0.073	1															
**item 6**	0.519	0.388	0.573	-0.086	0.487	1														
**item 7**	0.369	0.332	0.396	-0.071	0.484	0.493	1													
**item 8**	-0.163	-0.111	-0.230	0.477	-0.182	-0.236	-0.202	1												
**item 9**	0.326	0.203	0.404	-0.099	0.311	0.448	0.362	-0.241	1											
**item 10**	0.369	0.239	0.423	-0.095	0.384	0.470	0.353	-0.182	0.394	1										
**item 11**	0.259	0.274	0.237	0.054	0.283	0.338	0.292	-0.061	0.196	0.237	1									
**item 12**	-0.322	-0.227	-0.370	0.342	-0.305	-0.436	-0.290	0.481	-0.291	-0.285	-0.202	1								
**item 13**	0.314	0.260	0.351	-0.150	0.371	0.441	0.318	-0.197	0.298	0.325	0.189	-0.286	1							
**item 14**	0.341	0.270	0.454	-0.094	0.373	0.531	0.348	-0.236	0.417	0.436	0.249	-0.342	0.425	1						
**item 15**	0.272	0.208	0.312	-0.118	0.296	0.331	0.264	-0.173	0.295	0.328	0.125	-0.217	0.311	0.354	1					
**item 16**	-0.275	-0.199	-0.341	0.361	-0.293	-0.374	-0.272	0.510	-0.288	-0.289	-0.140	0.632	-0.283	-0.340	-0.235	1				
**item 17**	0.317	0.250	0.360	-0.081	0.266	0.401	0.284	-0.162	0.310	0.365	0.208	-0.231	0.301	0.350	0.239	-0.201	1			
**item 18**	0.460	0.334	0.511	-0.093	0.430	0.720	0.422	-0.235	0.440	0.453	0.318	-0.451	0.412	0.519	0.342	-0.373	0.416	1		
**item 19**	0.299	0.224	0.352	-0.135	0.300	0.350	0.287	-0.185	0.329	0.338	0.137	-0.249	0.316	0.387	0.661	-0.265	0.260	0.387	1	
**item 20**	0.388	0.353	0.436	-0.106	0.536	0.581	0.529	-0.239	0.388	0.400	0.303	-0.395	0.429	0.472	0.329	-0.369	0.286	0.556	0.372	1
