# Supplementary material for: The Psychometric Properties of the Center for Epidemiologic Studies Depression Scale in Chinese Primary Care Patients: Factor Structure, Construct Validity, Reliability, Sensitivity and Responsiveness
Source: PLoS One. 2015 Aug 7;10(8):e0135131. doi: 10.1371/journal.pone.0135131 (PMC4529142; doi:10.1371/journal.pone.0135131)
Supplement: S1 Fig — (PDF) [file pone.0135131.s002.pdf]

S1 Figure. Subject Recruitment Flowchart

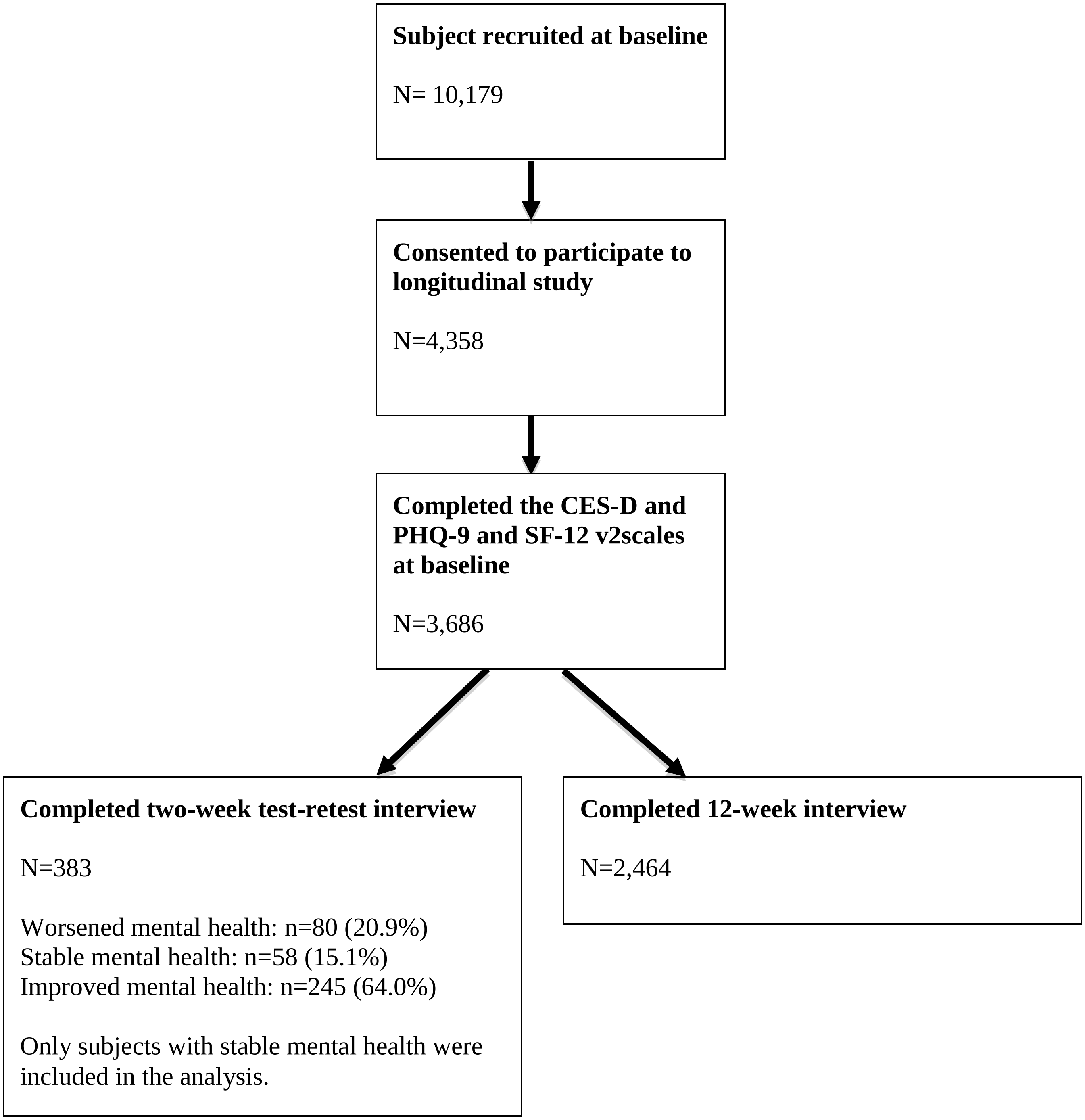

CES-D: Center for Epidemiologic Studies Depression Scale
PHQ-9: Patient Health Questionnaire -9
