# Supplementary material for: The Psychometric Properties of the Center for Epidemiologic Studies Depression Scale in Chinese Primary Care Patients: Factor Structure, Construct Validity, Reliability, Sensitivity and Responsiveness
Source: PLoS One. 2015 Aug 7;10(8):e0135131. doi: 10.1371/journal.pone.0135131 (PMC4529142; doi:10.1371/journal.pone.0135131)
Supplement: S2 Fig — The CES-D and PHQ-9 were sensitive enough to detect difference between the subject, with an AUC >0.7 for all instruments. (PDF) [file pone.0135131.s003.pdf]

S2 Fig

The sensitivity of the CES-D and the PHQ-9 to differentiate subjects with depression and those without depression

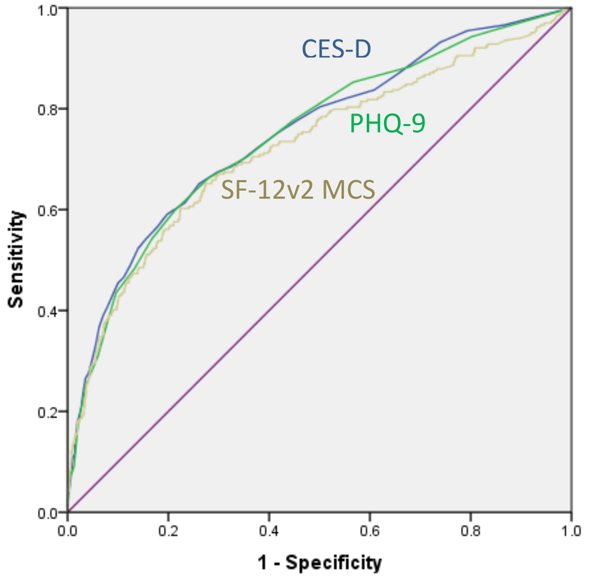

The figure shows the ROC curve for sensitivity analysis. The CES-D, PHQ-9 and SF-12 v2 MCS were sensitive enough to detect difference between the subject, with an AUC >0.7 for all instruments.
