# Supplementary material for: The Psychometric Properties of the Center for Epidemiologic Studies Depression Scale in Chinese Primary Care Patients: Factor Structure, Construct Validity, Reliability, Sensitivity and Responsiveness
Source: PLoS One. 2015 Aug 7;10(8):e0135131. doi: 10.1371/journal.pone.0135131 (PMC4529142; doi:10.1371/journal.pone.0135131)
Supplement: S3 Fig — With the standard of the AUC>0.7, the CES-D (AUC = 0.75) but not the PHQ-9 (AUC = 0.64) was adequate to differentiate subjects who improved and those with stable or worsened depressive symptoms. (PDF) [file pone.0135131.s004.pdf]

S3 Fig

The external responsiveness of the CES-D and the SF-12 v2 MCS

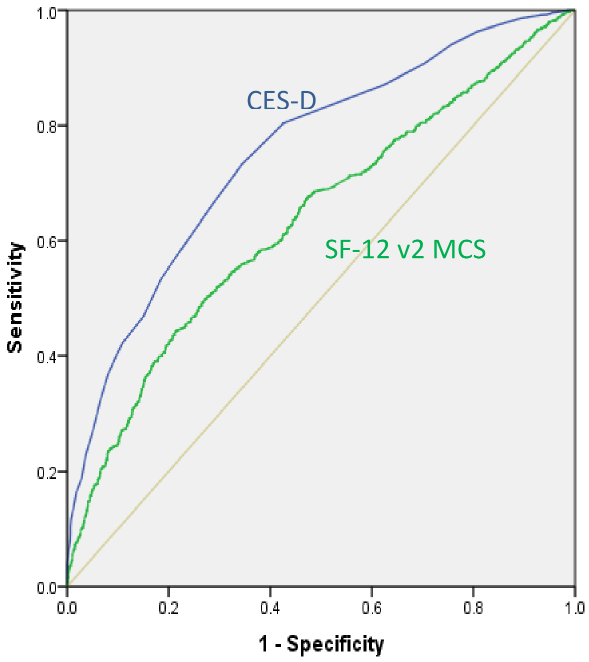

The figure shows the ROC curve for sensitivity analysis. With the standard of the AUC>0.7, the CES-D (AUC = 0.75) but not the PHQ-9 (AUC = 0.64) was adequate to differentiate subjects who improved and those with stable or worsened depressive symptoms
