# Supplementary material for: The Psychometric Properties of the Center for Epidemiologic Studies Depression Scale in Chinese Primary Care Patients: Factor Structure, Construct Validity, Reliability, Sensitivity and Responsiveness
Source: PLoS One. 2015 Aug 7;10(8):e0135131. doi: 10.1371/journal.pone.0135131 (PMC4529142; doi:10.1371/journal.pone.0135131)
Supplement: S1 Instrument — (PDF) [file pone.0135131.s005.pdf]

S1 Instrument

<u>流行病學研究中心抑鬱量表</u>
<u>The Center for Epidemiologic Studies Depression Scale (CES-D)</u>

以下句子描述一些自我感覺或行為。請圈出最接近您<u>**過去一週**</u>的狀況。

Below is a list of some of the ways you may have felt or behaved. Please indicate how often you have felt this way during the **past week**: (*circle **one** number on each line*)

<u>**過去一週**</u><br>**During the past week...**	**很少或完全沒有**<br>**Rarely or none of the time**<br>（少過 1 日）<br>(Less than 1 day)	**有幾天**<br>**Some or a little of the time**<br>（持續 1-2 日）<br>(1-2 days)	**間中或一半時間**<br>**Occasional or moderate amount of time**<br>（持續 3-4 日）<br>(3-4 days)	**經常或近乎每天**<br>**Most or all of the time**<br>（持續 5-7 日）<br>(5-7 days)
1. 我被一些平時不會困擾我的事情困擾<br>I was bothered by things that usually don’t bother me	0	1	2	3
2. 我不想吃東西，我的胃口很差<br>I did not feel like eating; my appetite was poor	0	1	2	3
3. 即使有家人和朋友的幫忙，我仍然覺得憂鬱<br>I felt that I could not shake off the blues even with help from my family	0	1	2	3
4. 我覺得我不比其他人差<br>I felt that I was just as good as other people	0	1	2	3
5. 我很難集中精神工作<br>I had trouble keeping my mind on what I was doing	0	1	2	3
6. 我覺得情緒低落<br>I felt depressed	0	1	2	3
7. 我覺得我做每件事情都很吃力<br>I felt that everything I did was an effort	0	1	2	3
8. 我對將來抱有希望<br>I felt hopeful about the future	0	1	2	3
9. 我覺得自己一生很失敗<br>I thought my life had been a failure	0	1	2	3

10. 我覺得恐懼<br>I felt fearful	0	1	2	3
11. 我睡眠不安寧<br>My sleep was restless	0	1	2	3
12. 我很開心<br>I was happy	0	1	2	3
13. 我比平時少說話<br>I talked less than usual	0	1	2	3
14. 我覺得孤獨<br>I felt lonely	0	1	2	3
15. 我覺得其他人不友善<br>People were unfriendly	0	1	2	3
16. 我很享受生活<br>I enjoyed life	0	1	2	3
17. 我會經常無故哭泣<br>I had crying spells	0	1	2	3
18. 我覺得不開心<br>I felt sad	0	1	2	3
19. 我覺得其他人不喜歡我<br>I felt that people disliked me	0	1	2	3
20. 我提不起勁<br>I could not get "going"	0	1	2	3
